# Regional citrate anticoagulation with a low-concentration solution in predilution-postdilution CVVH

**DOI:** 10.1186/cc10974

**Published:** 2012-03-20

**Authors:** V Pistolesi, S Morabito, L Tritapepe, L Cibelli, M Ambrosino, F Polistena, L Zeppilli, E Strampelli, MI Sacco, A Pierucci

**Affiliations:** 1 Policlinico Umberto I, Rome, Italy

## Introduction

Systemic anticoagulation (AC) can increase the bleeding risk in CRRT. However, regional citrate anticoagulation (RCA) is a valid alternative to heparin (Hep) in patients at high risk of bleeding. The aim was to evaluate efficacy and safety of RCA-CVVH using a low-concentration citrate (Citr) solution.

## Methods

In cardiac surgery patients with AKI we adopted RCA-CVVH as an alternative to Hep or no-AC CRRT. Criteria for switching to RCA: early circuit clotting (24 hours) or Hep-related complications. RCA-CVVH was performed with a predilution Citr solution (12 mmol/l) and a postdilution hemofiltration solution (HCO_3_^- ^32 mEq/l). In relation to blood flow rate (Qb), the Citr solution rate was set to meet a circuit Citr concentration of 3 mmol/l and modified to obtain circuit Ca^2+ ^< 0.4 mmol/l. CaCl_2 _(10%) was infused to maintain systemic Ca^2+ ^(s-Ca^2+^) of 1.1 to 1.25 mmol/l. To facilitate CVVH settings, we developed a mathematical model to estimate the metabolic Citr load, buffer balance and Ca^2+ ^loss.

## Results

In 30 patients at high bleeding risk (age 70.5 ± 9.3, SOFA score 13.7 ± 2.5) the AC modality was switched to RCA-CVVH from no AC or Hep. CVVH initial settings: dialysis dose 33.6 ± 3.4 ml/kg/hour; Qb 135 ± 14 ml/minute; Q Citr 1,703 ± 250 ml/hour; Q postdilution 761 ± 181 ml/hour; Citr load 11.6 ± 2.1 mmol/hour; CaCl_2 _3.7 ± 1.5 ml/hour. Target circuit Ca^2+ ^and s-Ca^2+ ^were maintained (0.37 ± 0.09 and 1.18 ± 0.13 mmol/l) with few modifications of Citr and CaCl_2 _infusion rates. We used 146 RCA-CVVH circuits with filter life 50.5 ± 35.8 hours (median 41; total 7,372). RCA-stopping causes: 34% CVC malfunction, 24% alarm handling/technical issues, 20% scheduled, 14% medical procedures, 8% others. Before starting RCA, we used 69 Hep circuits (2,015 hours) and 74 no-AC circuits (1,827 hours) with a filter life of 29.2 ± 20.7 hours (median 22) and 24.7 ± 20.6 hours (median 20), shorter than RCA (*P *< 0.0001). Circuits running at 24, 48 and 72 hours (%): RCA 73, 42 and 28; Hep 43, 23 and 10; and no-AC 38, 12 and 5 (log-rank test *P *< 0.0001). During RCA-CVVH no patients had bleeding complications and the transfusion rate was lower if compared to other AC modalities (0.29 vs. 0.69 blood units/day, *P *= 0.001). PLT count (*P *= 0.018) and AT-III activity (*P *= 0.009) increased throughout days of RCA, reducing supplementation needs. RCA has been stopped for Citr accumulation in one patient (calcemia/s-Ca^2+ ^>2.5).

## Conclusion

In this experience, RCA allowed one to safely prolong the filter life, decreasing the transfusion rate and supplementation needs for AT-III and PLT. The use of a mathematical model allowed one to simplify the CVVH settings. Therefore, RCA should be worthy of more consideration as the first-choice CRRT AC modality in patients at high risk of bleeding.

